# Neural Basis of Moral Elevation Demonstrated through Inter-Subject Synchronization of Cortical Activity during Free-Viewing

**DOI:** 10.1371/journal.pone.0039384

**Published:** 2012-06-20

**Authors:** Zoë A. Englander, Jonathan Haidt, James P. Morris

**Affiliations:** Department of Psychology, University of Virginia, Charlottesville, Virginia, United States of America; Charité University Medicine Berlin, Germany

## Abstract

**Background:**

Most research investigating the neural basis of social emotions has examined emotions that give rise to negative evaluations of others (e.g. anger, disgust). Emotions triggered by the virtues and excellences of others have been largely ignored. Using fMRI, we investigated the neural basis of two “other-praising" emotions – Moral Elevation (a response to witnessing acts of moral beauty), and Admiration (which we restricted to admiration for physical skill).

**Methodology/Principal Findings:**

Ten participants viewed the same nine video clips. Three clips elicited moral elevation, three elicited admiration, and three were emotionally neutral. We then performed pair-wise voxel-by-voxel correlations of the BOLD signal between individuals for each video clip and a separate resting-state run. We observed a high degree of inter-subject synchronization, regardless of stimulus type, across several brain regions during free-viewing of videos. Videos in the elevation condition evoked significant inter-subject synchronization in brain regions previously implicated in self-referential and interoceptive processes, including the medial prefrontal cortex, precuneus, and insula. The degree of synchronization was highly variable over the course of the videos, with the strongest synchrony occurring during portions of the videos that were independently rated as most emotionally arousing. Synchrony in these same brain regions was not consistently observed during the admiration videos, and was absent for the neutral videos.

**Conclusions/Significance:**

Results suggest that the neural systems supporting moral elevation are remarkably consistent across subjects viewing the same emotional content. We demonstrate that model-free techniques such as inter-subject synchronization may be a useful tool for studying complex, context dependent emotions such as self-transcendent emotion.

## Introduction

Scientific investigations of emotion typically focus on the original six “basic" emotions first described by Ekman and Friesen [1]. These emotions – happiness, sadness, anger, fear, surprise and disgust – each have a distinctive cross-culturally recognizable facial expression and can be easily elicited in the lab. However, emotional experience is not limited to these six emotions. Ekman himself has long said that there are many more emotions that should be studied, only a few of which may turn out to have distinctive facial signals or clear analogues in other animals [2].

Most emotions involve an evaluation–a rapid appraisal of a person, action, or event as either good or bad [3]. There have been many studies of emotions that give rise to the negative evaluations of others (anger, disgust, and contempt), and of the self (shame and guilt). However, research on the positive social emotions appears much less in the literature than research on the negative emotions [4].

Recent work by Haidt and colleagues has sought to demonstrate the existence of a class of emotions they call “other-praising" emotions. These emotions are elicited by a positive appraisal of another person’s actions. They generally motivate people to praise others to third parties [5]–[6]. This class of emotions includes moral elevation, which is defined as a reaction to witnessing acts of moral beauty. Moral elevation has been found to motivate people to want to emulate the virtuous role model and do virtuous deeds themselves [6]–[7]. Another “other-praising" emotion is admiration. Because the word “admiration" is a broad term that is sometimes used to refer to admiration for moral virtue (which would overlap with elevation), we restrict the meaning of admiration to mean the positive emotional response to witnessing another person exceed normal standards of skill or talent. Admiration for skills/talents has been shown to be quite distinct from moral elevation. For example, Algoe and Haidt [5] found that admiration was generally reported by participants to be energizing. Admiration motivates people to work harder on their *own* goals and projects, and it was shown to be more likely than elevation to elicit self-reported feelings of chills or tingling in the skin. (The “other-praising" emotions also include the emotion of gratitude, which we will not discuss because it cannot be elicited by a video. Therefore, it cannot be studied using the methods we describe below.).

Elevation and admiration are unusual among emotions because they are not elicited by events directly relevant to the self. Cognitive theories of emotion typically categorize emotions based on the appraisals that trigger them. As Lazarus [8] put it, an appraisal is “an evaluation of the significance of knowledge about what is happening for our personal well-being. Only the recognition that we have something to gain or lose, that is, that the outcome of a transaction is relevant to goals and well-being, generates an emotion" [3], [9]. Therefore, it is puzzling–and it requires some investigation– to understand how and why people can experience chills, and can be moved to tears, just by seeing other people do extraordinary things with no implications for the self. In fact, the “other-praising" emotions are sometimes referred to as “self-transcendent" emotions because they seem to take people out of their ordinary everyday focus on their own goals and projects. These emotions make people feel “uplifted" or “inspired" [10].

Techniques that have been successful in identifying the neural underpinnings of the basic emotions (such as the use of standardized still photographs to elicit emotions) are likely to be less successful when studying the “other-praising emotions". Unlike basic emotions such as fear and disgust, elevation is generally induced by narratives with plotlines that build slowly. It cannot be induced by a simple photograph (e.g., of a person giving money to a beggar). While admiration can perhaps be induced by a photograph, it too is generally enhanced by a story that sets up the background and the challenge that the admirable person overcame. Therefore, these emotions present a unique challenge: how can we present stimuli with enough context to evoke them, and how can we design an analysis strategy that is compatible with such complex stimuli?

Due to these difficulties, there were no documented empirical attempts to characterize the neural basis of these emotions until very recently. In an fMRI study, Immordino-Yang and colleagues [11] elicited admiration and compassion across four different categories – admiration for virtue (which we call “moral elevation"), admiration for skill, compassion for social/psychological pain and compassion for physical pain. The authors relied on a “reminder" approach to present the emotional stimuli. This approach involved rigorously pre-testing each emotion by allowing participants to view full-length videos before entering the scanner. While in the scanner, participants viewed reminders of the stimuli while attempting to self-induce a similar emotional state to that experienced in pre-testing. Using this approach, the authors were able build a temporal model of the expected BOLD response necessary for traditional fMRI analytic techniques (general linear model analysis) while also using complex stimuli to evoke the target emotion. Compelling evidence for the role of brain systems supporting interoceptive processes in the subjective experience of admiration and compassion was presented. Unfortunately, the study only reported results for pairs of conditions merged together, with each “admiration" condition being paired with a “compassion" condition, which doesn’t allow for distinctions between moral elevation and admiration for skill. Furthermore, the “reminder" experimental approach does not allow for a direct measure of the emotional experience as it is naturally evoked, rather it asks the subject to re-induce an emotional state that was experienced in the past.

Due to the limitations associated with applying typical fMRI experimental design and analysis procedures to study slow-building emotional experience, we turned to a non-traditional approach that has been previously successful in identifying neural systems involved in shared sensory experience. Specifically, Hasson and colleagues [12] have developed an analytic technique where the BOLD response in an individual brain is used to model the activity in another brain. By applying this inter-subject correlation procedure, they have demonstrated extensive voxel-to-voxel synchrony between individuals freely-viewing the same movie. This approach has provided fascinating evidence for how individuals process complex audio- visual stimuli in the same way (how individuals *see* the world in the same way) and has led us to consider its utility in understanding to what degree humans *feel* the world in the same way, or at least during the experience of “other-praising" emotions.

In the present study we adopted this inter-subject correlation approach to characterize neural systems supporting moral elevation and admiration. This analytic approach allowed us to use a free-viewing paradigm where video clips were presented to subjects in the scanner. These video clips have been demonstrated to be effective in eliciting the target emotions. We predicted that a high-degree of synchrony in the BOLD signal across individuals would be induced during the experience of “other-praising"emotion. We hypothesized that the inter-subject synchronization would be especially pronounced in brain regions involved with interoceptive processes and self-referential thought, as well as in the insula, as was shown by Immordino-Yang and colleagues [4], [11].

## Methods

### Selection of Stimuli

The video clips used in this study were selected from a collection of YouTube clips previously validated by a study on www.YourMorals.org. The clips were shown to effectively elicit the target emotions. In a large and diverse population. 2,815 participants were shown one of 30 short video clips (10 candidate clips for each of the three emotions; elevation, admiration, and neutral). To assess the distinct characteristics of each video, a web survey was created that asked about each of the main components of the emotional content of the video, as well as a few additional questions about the participants’ overall response to the video. We selected the videos to be between 3 and 5 minutes in length to ensure that large variations in duration wouldn’t confound our analysis.

Of the videos that fit the criteria for length, we chose three videos rated highly for ‘moral good’ and ‘positive emotion’ for the moral elevation videos. One video, for example, was the story of the “subway hero", a New York construction worker who endangered himself to save, a young man who had suffered a seizure and fallen onto the tracks, from being struck by a New York City subway train.

Additionally, we chose three videos rated highly for ‘great skill or talent’ and ‘positive emotion’ for our admiration for physical skill videos. One video, for example, showed the highlights of a professional basketball player’s career. Finally, we selected two control videos that were rated as interesting but did not contain content rated as morally good or depicting great skill. As an example, one video was an informational clip about how a flute is constructed. See Table 1 for more information about each of the videos.

**Table 1:** Video Stimuli.

	Plot Summary	Duration
**Moral Elevation**		
EL1	A young man who was born blind and crippled learns to play the piano beautifully and is able to participate in a marching band.	303 s
EL2	A boy with autism participates in a basketball game.	130 s
EL3	A man saves a stranger’s life on the New York City subway.	124 s
**Admiration**		
AP1	A child sings somewhere over the rainbow for a talent competition.	177 s
AP2	Dancers compete on a talent competition.	192 s
AP3	A hall of fame basketball player’s career.	246 s
**Neutral**		
N1	Interview with an actress about her TV show.	120 s
N2	Interview on talk show.	256 s
N3	Tutorial about how a flute is constructed	299 s

### fMRI Experiment

#### Participants

In the fMRI experiment, 10 healthy volunteers (age 20 to 27, 7 female, 3 male) participated. All volunteers had normal or corrected-to-normal vision, and were screened against neurological and psychiatric diseases. Volunteers provided written informed consent prior to participation.

#### Stimuli

Each participant watched the same nine video clips, three belonging to the moral elevation condition, three belonging to the admiration condition, and three belonging to the neutral condition (See Table 1). The order of stimulus presentation was counterbalanced across participants such that videos were presented in three blocks, with one video clip of each condition in each block. The last video clip of each block belonged to the neutral condition. One of the video clips in the neutral condition was not analyzed because the scanner stopped before the narrative had concluded for some of the subjects. Therefore, all of the analysis contains only two examples of neutral videos. Subjects were also instructed to lie still with their eyes closed while no visual or auditory stimuli were presented during one scan three minutes in length. This data was used to identify areas of correlation that may have been introduced by scanner noise or data processing procedures, as no relevant correlation is expected in the absence of a stimulus.

Using Psychophysics toolbox for MATLAB, the stimuli were presented using an LCD projector (AVOTEC) that projected images onto a screen located behind the subject’s head. Participants viewed the stimuli through a mirror attached to the head coil, and listened to the audio through headphones. Participants were not told to induce a specific emotional state, they were told only to attend to the stimuli as they were presented. During the resting state scan, participants were asked to lie still with their eyes closed. Timing of the video clips was synchronized to the TTL pulse received with the acquisition of the first TR.

#### Imaging

Scanning was performed on a Siemens 3 Tesla MAGNETOM Trio with a 12-channel head coil. 176 high-resolution T1 weighted images were acquired using Siemens’ MPRAGE pulse sequence (TR, 1900 ms; TE, 2.53 ms; FOV, 250 mm; voxel size, 1 mm×1 mm×1 mm) and used for co-registration with functional data. Whole-brain functional images were acquired using a T2* weighted EPI sequence (repetition time = 2000 ms, echo time = 40, FOV = 192 mm, image matrix = 64×64, voxel size = 3.0×3.0 × 4.2 mm; flip angle = 90°, 28 axial slices).

#### Pre-Processing

FMRI data processing was carried out using FEAT (FMRI Expert Analysis Tool) Version 5.98, part of FSL (FMRIB’s Software Library, www.fmrib.ox.ac.uk/fsl). Motion was detected by center of mass measurements implemented using automated scripts developed for quality assurance purposes and packaged with the BXH/XCEDE suite of tools, available through the Bioinformatics Information Research Network (BIRN). Participants that had greater than a 3-mm deviation in the center of mass in the x-, y-, or z-dimensions were excluded from further analysis. The following pre-statistics processing was applied; motion correction using MCFLIRT [13], slice-timing correction using Fourier-space time-series phase-shifting; non-brain removal using BET [14], spatial smoothing using a Gaussian kernel of FWHM 8.0 mm; grand-mean intensity normalization of the entire 4D dataset by a single multiplicative factor; high-pass temporal filtering (Gaussian-weighted least-squares straight line fitting, with sigma = 50.0 s). Additionally, each functional volume was registered to the participant’s anatomical image, and then to the standard Montreal Neurologic Institute (MNI) template brain using FLIRT [13]. Each individual anatomical image was also registered to standard space and segmented into gray matter, white matter, and CSF components using FAST [15]. The preprocessed data were then used in the analysis procedures described below.

#### Inter-subject correlation

The primary analysis followed the methods of Hasson and colleagues [12], and consisted of an assessment of the temporal synchronization in the BOLD signal between different individuals’ brains that occurred in response to the stimuli. A voxel should show a high degree of correlation with a corresponding voxel in another brain if the two time-courses show similar temporal dynamics, time-locked to the stimuli. As demonstrated by Hasson and colleagues, extensive synchronization is observed in visual and auditory regions as participants freely view complex stimuli. However, no inter-subject synchronization would be expected in data sets where the participants were scanned in the absence of stimuli. Inter-subject temporal correlation would not be expected in the situation where there is no stimulus to induce the time-locking of the neural response. We extended this methodology beyond the study of visual and auditory processing, to the investigation of the experience of “other-praising" emotions.

In order to quantify the degree of synchronization in the BOLD signal between corresponding voxels in different individuals’ brains, the time course of each voxel in a template brain was used to predict activity in a target brain, resulting in a map of correlation coefficients. Using the segmented and standardized anatomical images, we restricted this procedure only to voxels that were classified as gray matter in both the template and target brains. Overall, there were 45 pair-wise comparisons for each video clip and the resting state run between 10 individuals. After maps of correlation coefficients were generated for each pair, the correlation maps were concatenated into a 4-D data set (x×y×z×correlation coefficient for each pair). To determine which voxels showed overall correlation across all pair-wise comparisons, a non-parametric permutation method as implemented by FSL randomise was used for thresholding and correction for multiple comparisons using FWE (family-wise error) correction [16]. This resulted in a single image for each video clip describing which voxels have correlation coefficients that are significantly different from zero with p<0.05. This method of determining probability was used because the null distributions for these datasets were assumed to be non-normal.

### Peak-Moment Video Ratings

In order to establish the portions of the movie clips that were most likely to evoke strong emotions, we conducted a separate behavioral study intended to provide moment-by-moment ratings of positive and negative emotion for each of our video clips. We were looking to establish which portions of the video clips individuals found to be most emotionally arousing. Twenty-one volunteers (age 18–32, 13 females) who did not previously participate in the fMRI portion of the experiment participated in a behavioral rating experiment. In this experiment, the participants moved a slider up and down to reflect positive or negative feelings while viewing the videos. Participants controlled the slider with arrow keys on a computer keyboard and were instructed to move it up when feeling positive and to move it down when feeling negative. Twenty-five data points per second were collected on an arbitrary scale that ranged from −500 (most negative) to 500 (most positive). From this study we were able to generate a time-series for each participant of moment-by-moment video ratings. We then averaged and normalized the ratings time-course for each video across participants in order to identify “peak-moments" of positive ratings for each of the videos. Peak moments were identified as windows around areas of the time-course in which the normalized rating exceeded z = 2.3. The average length of the peak moments was 10 TRs (20 seconds). There were no windows in the normalized time-courses of the neutral videos that met the criteria for a “peak moment."

## Results

**Figure 1:**
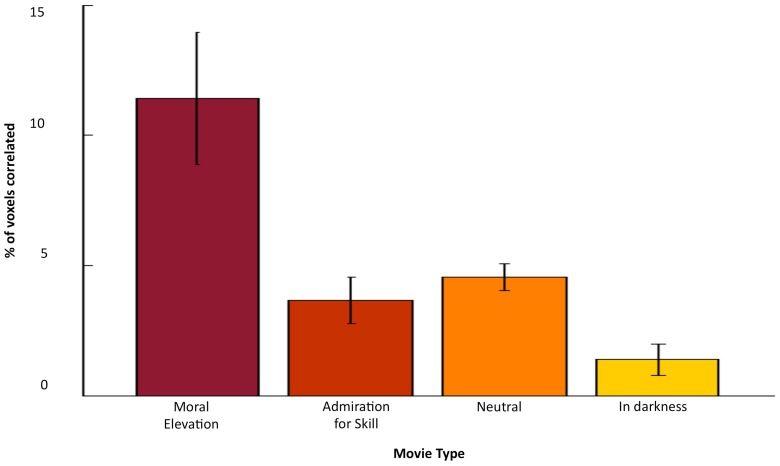
Percentage of correlated gray matter voxels for each condition.

**Figure 2:**
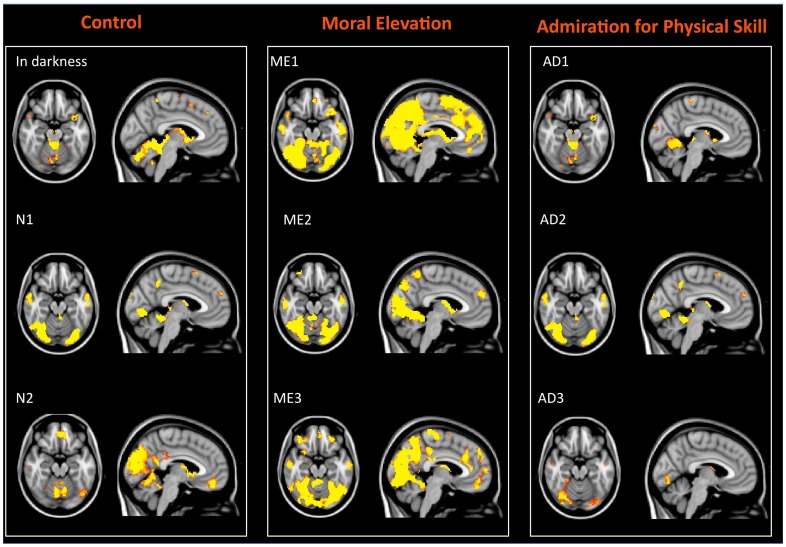
Significantly correlated voxels across all pair-wise comparisons for each condition. Color maps indicate the significance level at each voxel (*p*<.05–*p*<1×10^−3^).

We observed extensive regions of gray matter that showed temporal synchronization in the BOLD signal between brains for all video clips. In contrast, very little synchronized activity was observed in the resting-state data. This result is demonstrated in Figure 1 and Table 2 show the mean percentage of gray matter voxels in the brain for each of the three types of video clips and in the resting state that fit the following criteria: (i) the correlation coefficient of that voxel was greater than 0.2 in an individual pair-wise correlation map, (ii) and that voxel showed a significant (P<0.05) correlation across all 45 pair-wise comparisons Voxels that fit these criteria were considered to be temporally synchronized between brains. Overall, the elevation clips induced a greater amount of inter-subject temporal synchronization across all gray matter voxels relative to the other three conditions. Figure 2 shows the extent of the significantly correlated BOLD signal for each video clip.

**Table 2:** Percentage of correlated gray matter voxels for each condition.

	Mean % correlated	Std Dev.
Elevation	11.41	2.55
Admiration	3.65	0.89
Neutral	4.54	0.51
In darkness	1.39	0.61

Next, we found which voxels responded to the experience of an other-praising emotion, regardless of whether it was elevation or admiration. For this analysis, we created a mask of voxels that were significantly correlated for both the elevation and admiration conditions, but not the neutral condition. The results revealed small clusters of voxels within the bilateral middle temporal gyrus and fusiform gyrus that showed significant inter-subject synchronization across all pair-wise correlation maps for both elevation and admiration (Table 3).

**Table 3:** Clusters of voxels correlated in both the admiration for physical skill and moral elevation conditions.

Anatomical Region	k	Hem	x	y	z
Middle Temporal Gyrus	262	R	59.8	−46.8	5
	18	L	−58.2	−40.6	−4
Occipital Pole	64	L	−11	−97.6	−9
Fusiform Gyrus	34	R	19.6	−90.2	−18.4

X,Y,Z coordinates reflect the center of gravity of the cluster in MNI space.

**Figure 3:**
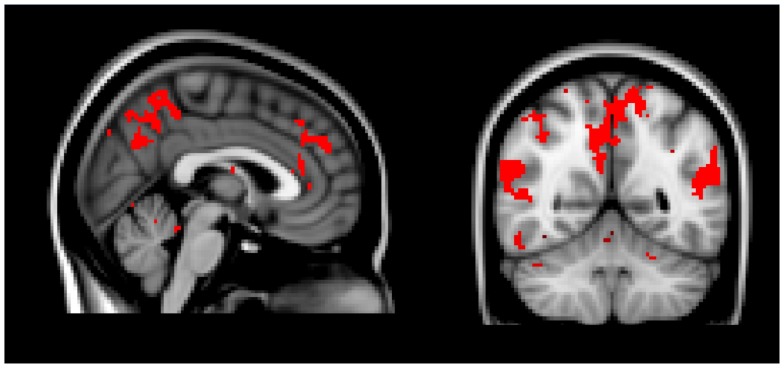
Mask of voxels significantly correlated in all moral elevation videos, but not correlated during admiration for physical skill or neutral videos.

To isolate voxels that responded uniquely to elevation, we created a mask of voxels that displayed significant correlation across participants in all three elevation videos, but not any of the neutral or admiration videos. From this analysis, we identified clusters of voxels in the anterior and posterior cingulate, precuneus, bilateral temporoparietal junction, superior frontal gyrus, inferior frontral gyrus and angular gyrus that appeared to be responding consistently and uniquely to the elevation videos (Table 4; Figure 3). We classified and reported each cluster of voxels based on its center of gravity, however the clusters identified as the angular gyrus in the left hemisphere and the superior frontal gyrus in the right hemisphere include the bilateral parietal operculum.

**Table 4:** Clusters of voxels significantly correlated in all moral elevation video clips but none of the other clips.

Anatomical Region	k	Hem	x	y	z
Precuneus	3147	R/L	26.2	−61.4	37.4
Angular Gyrus	402	L	−51.2	−57	21.8
Superior Frontal Gyrus	305	R	27.2	4.8	57.8
Frontal Pole	175	R	38	47.8	−18.4
Anterior Cingulate Gyrus	166	R	4.6	40.4	26.8
Fusiform Gyrus	136	L	−14.2	−81.6	−20.8
Inferior Frontal Gyrus	133	R	52.6	12.2	20.6
Posterior Cingulate	53	L	−9.4	−35.2	39.6

X,Y,Z coordinates reflect the center of gravity of the cluster in MNI space.

**Figure 4:**
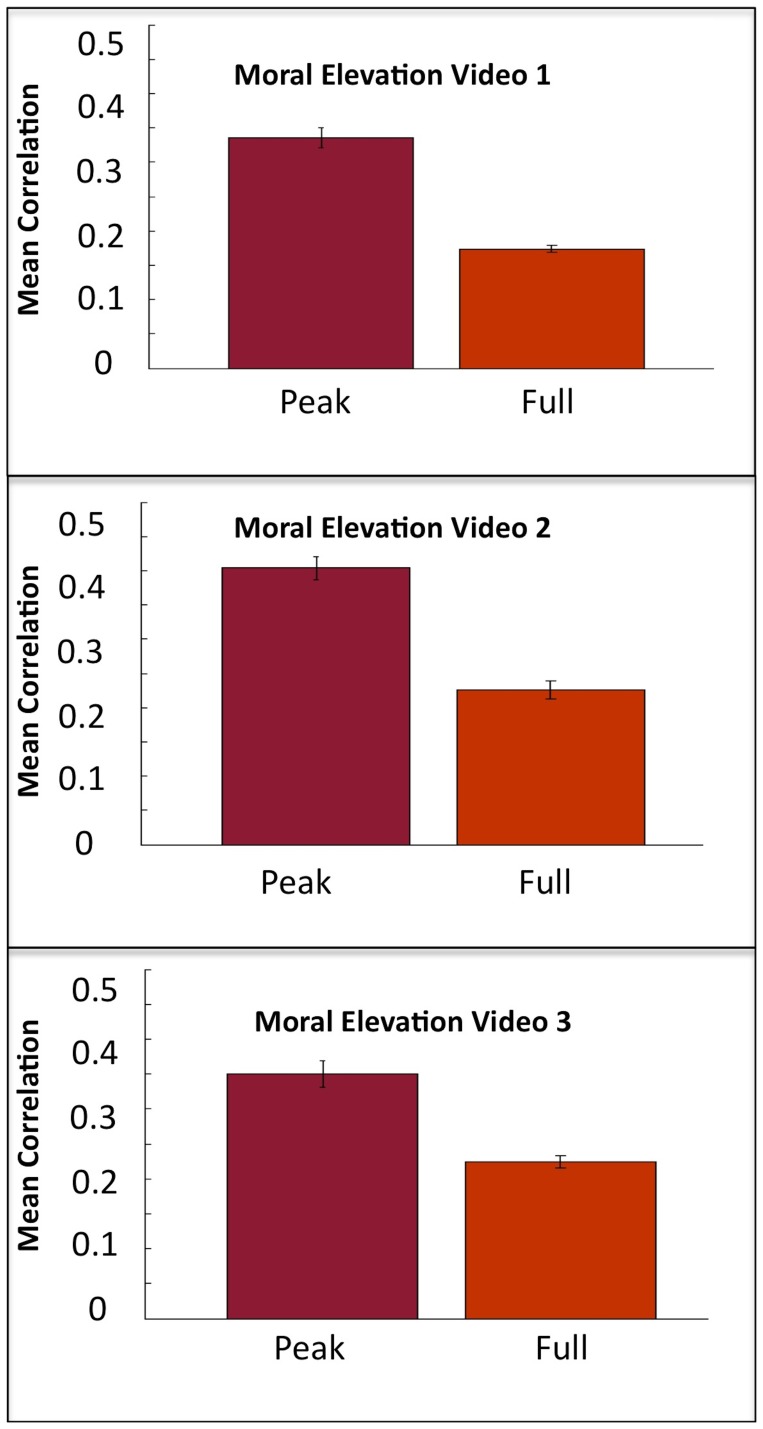
Average correlation of gray matter voxels during peak moments vs. whole videos for the three moral elevation videos.

In order to demonstrate that the high inter-subject synchronization evoked by the elevation videos was related to feelings of positive emotion; we conducted an inter-subject synchronization analysis on the peak moments of the video, the moments that were rated most positive during the behavioral study described previously in this manuscript. For each of the three elevation videos, we repeated the inter-subject synchronization procedure to measure the average correlation coefficient within gray matter voxels during the peak moments. The average correlation coefficient was found to be higher during peak moments relative to the average correlation coefficient for the whole video for each of the three moral elevation (ME) videos (ME1–*t*_(44)_ = 11.94, *p<.*001; ME2–*t*_(44)_ = 11.37, *p<.*001; ME3–*t*_(44)_ = 9.128, *p<*.001) (Figure 4).

## Discussion

The current study was concerned with brain systems supporting feelings of moral elevation (admiration for moral virtue) and admiration for the physical skill or talent of others. These “other-praising" emotions appear to be catalysts for positive behavioral change [5], yet little is known about the brain mechanisms involved in the experience of these emotions. Unlike the basic emotions, the other-praising emotions cannot be easily elicited with static photographs displaying arousing content. Stimuli that are capable of evoking “other-praising" emotions are often complex and story-like, making the temporal model typically employed in fMRI analysis difficult to derive. In this study we used an inter-subject synchronization approach that has previously been used in fMRI studies featuring free-viewing of movies to demonstrate that audio-visual processing of complex stimuli occurs similarly across individuals. We adopted this approach to investigate the neural systems supporting the emotional response to excellence in others, specifically to test whether neural activity associated with moral elevation is different than the admiration for physical skill.

Using the inter-subject synchronization technique, we discovered a striking amount of correlation in the BOLD signal across individuals. Replicating Hasson and colleagues [12], we observed extensive correlation in the audio-visual cortex when participants freely-viewed video clips, regardless of the emotional content. Elevating videos were especially effective in eliciting high synchronization between subjects. Along with the audio-visual cortex, these videos produced significant correlation in anterior and posterior cingulate, precuneus, bilateral temporoparietal junction, superior frontal gyrus, inferior frontal gyrus, angular gyrus and insula. In contrast, admiration videos did not elicit synchrony in the BOLD signal as consistently, with the exception of those brain regions associated with audio-visual processing, and synchrony in the insula was observed for one of the admiration videos.

In a subsequent analysis, we found voxels that were significantly correlated in all moral elevation video clips, but not in any of the other video clips. From this analysis, we found that regions in the anterior and posterior cingulate, precuneus, bilateral temporoparietal junction, superior frontal gyrus, inferior frontal gyrus, angular gyrus, and bilateral areas of the parietal operculum uniquely responded in the elevation condition. While many of the regions detected by this analysis are often implicated in “default" mode processing, it should be noted that the intersubject synchronization technique is not optimized to detect intrinsic connectivity *within* participants, but rather correlations *between* participants during stimulation. Therefore, activity in these regions is likely related to processing of the content of the video.

Our findings closely parallel those found in a recent study by Immordino-Yang and colleagues. Here, the authors used brief narratives designed to evoke admiration and compassion for others. They report involvement of the posterior cingulate, retrosplinial cortex and precuneus – referred to as the posteromedial cortices (PMC) – when subjects experienced either admiration for the virtues of others or compassion for others’ psychological pain [11]. However, these same areas are not involved in the admiration for physical skill or compassion for physical pain. In their study, Immordino-Yang and colleagues used a recall paradigm where subjects were presented with emotional films before entering the scanner, shown refresher clips during scanning, and told specifically to induce the same emotional state as they experienced in the preparation session. Here we find a similar pattern of effects when subjects are presented with the emotional stimuli in the scanner and are not told to specifically attend to their own emotional state.

While we are unable to pinpoint the precise role of these highly synchronized brain regions evoked by elevation videos, our findings do provide some unique insight into the mental operations that may lead to feelings of elevation. First, regions uniquely associated with peak moments of elevation are often implicated in mentalizing behavior – that is, the ability to make inferences about the beliefs and mind states of others [17]–[18]. Second, midline structures including precuneus, posterior and anterior cingulate have been proposed to play a role in self-referential processes including constructing a social narrative, integrating emotional experience and action planning [19]–[21]. Furthermore, our moral elevation videos also evoked synchrony in bilateral parietal operculum, which is part of the secondary somatosensory cortex and thought to play a role in emotional experience. Because these same structures seem to support both self-referential processes and mentalizing abilities, some have speculated that one’s own self is modeled when making inferences about others – a process referred to as simulation. Our data suggest that moral elevation engages these same processes, leading to an interesting paradox. While moral elevation may involve feelings of self-transcendence – a *reduction* in attention to the self- the actual feeling of elevation is dependent upon an *increase* of self-referential processes.

A potential limitation of the current investigation is that we did not collect any behavioral data from the group of subjects that were scanned. Because we wanted to replicate procedures previously successful in evoking high inter-subject synchronization during free-viewing, we chose to allow our subjects to freely view the stimulus without an overt task. Furthermore, we also wanted to test if the same pattern of activation previously reported in brain regions supporting self-referential processes would replicate even when subjects were not explicitly told to attend to their own emotional state. Future studies would be well served to combine the two approaches featured here. That is, moment-by-moment ratings of emotion could be collected while subjects view the videos in the scanner and used as a predictor of BOLD activity in candidate brain regions. This type of analysis would give us further evidence that the regions we report in this study are directly related to positive aspects of moral elevation.

Overall, using this inter-subject correlation technique, we were able to present complex emotional stimuli to individuals undergoing fMRI. We demonstrated that a high degree of inter-subject correlation occurred in voxels responding to the emotional content of the videos, specifically within regions associated with interoceptive processing in the moral elevation condition.
